# Chelidonic Acid and Its Derivatives from *Saussurea Controversa*: Isolation, Structural Elucidation and Influence on the Osteogenic Differentiation of Multipotent Mesenchymal Stromal Cells In Vitro

**DOI:** 10.3390/biom9050189

**Published:** 2019-05-16

**Authors:** Elena Avdeeva, Elvira Shults, Tatyana Rybalova, Yaroslav Reshetov, Ekaterina Porokhova, Irina Sukhodolo, Larisa Litvinova, Valeria Shupletsova, Olga Khaziakhmatova, Igor Khlusov, Artem Guryev, Mikhail Belousov

**Affiliations:** 1 Department of Pharmaceutical Analysis, Siberian State Medical University, Tomsk 634050, Russia; ferroplex2013@yandex.ru (Y.R.); titan-m@mail.ru (A.G.); mvb63@mail.ru (M.B.); 2 Laboratory of Medicinal Chemistry, Novosibirsk Institute of Organic Chemistry, Siberian Branch, Novosibirsk 630090, Russia; schultz@nioch.nsc.ru; 3 Novosibirsk State University, 2 Pirogova St., Novosibirsk 630090, Russia; 4 Center of Spectral Investigations, Novosibirsk Institute of Organic Chemistry, Siberian Branch, Novosibirsk 630090, Russia; rybalova@nioch.nsc.ru; 5 Department of Morphology and General Pathology, Siberian State Medical University, Tomsk 634050, Russia; porohova_e@mail.ru (E.P.); staranie@mail.ru (I.S.); khlusov63@mail.ru (I.K.); 6 Basic Laboratory of Immunology and Cell Biotechnology, Immanuel Kant Baltic Federal University, Kaliningrad 236041, Russia; larisalitvinova@yandex.ru (L.L.); vshupletsova@mail.ru (V.S.); hazik36@mail.ru (O.K.); 7 Research School of Chemistry & Applied Biomedical Sciences, Tomsk Polytechnic University, Tomsk 634050, Russia

**Keywords:** *Saussurea controversa* DC (Asteraceae), chelidonic acid, 4-oxo-4*H*-pyran-2.6-dicarboxylic acid esters, X-ray diffraction analysis, human multipotent mesenchymal stromal cells

## Abstract

4-oxo-4*H*-pyran-2.6-dicarboxylic acid (chelidonic acid, ChA) in the native state and in the complex with calcium [Ca(ChA)(H_2_O)_3_], named saucalchelin (CaChA), was isolated from the extract of *Saussurea controversa* leaves for the first time for the *Asteraceae* family. The structure of ChA was determined by NMR, MS and confirmed by X-ray analysis of its monomethyl ester, and CaChA was described by IR, ICP-MS, CHN analysis. The yield of ChA and CaChA was 45 mg/g and 70 mg/g of extract, respectively. The osteogenic activity of ChA, n-monobutyl ester of chelidonic acid, and CaChA has been studied in vitro in a 21-day culture of human adipose-derived multipotent mesenchymal stromal cells (hAMMSCs) in a standard nutrient medium without osteogenic supplements. CaChA significantly stimulated the growth of cell mass and differentiation of hAMMSCs into osteoblasts with subsequent mineralization of the culture and it may be a promising substance for accelerating bone tissue regeneration and engineering.

## 1. Introduction

The genus *Saussurea DC* is of considerable interest among a variety of medical plants containing more than 400 species belonging to the *Asteraceae* family. Many of these plants are widely used by local communities living in the Far East, Siberia, Tibet, and Mongolia to treat diseases of liver, kidneys, gastrointestinal tract, and musculoskeletal system [1,2]. It has been discovered that an extract from *Saussurea controversa* (SC) leaves, both alone and in a mixture with antibiotics, significantly reduces inflammatory processes and increases immunoreactivity in a biological model of osteomyelitis [3]. Previously, we isolated five quercetin glycosides from the SC extract and established their chemical structures that are likely to stimulate granulopoiesis and lymphopoiesis in rat bone marrow and enhance regeneration processes in damaged bone tissue under conditions of experimental osteomyelitis [4]. In our continued studies we isolated the predominant components of the water-soluble part of active extract from SC. Fractional crystallization and column chromatography were used to obtain a derivative of γ-pyron chelidonic acid (ChA) in the native state and in the form of a complex with calcium, CaChA, named saucalchelin, for the first time for the genus *Saussurea* and *Asteraceae* family. Nuclear magnetic resonance spectroscopy (NMR), mass spectroscopy (MS), inductively coupled plasma mass spectrometry (ICP-MS), and X-ray analysis were used to determine the structure of ChA (**1**); its monomethyl (**2**) and n-monobutyl (**3**) esters, which were obtained as a result of synthesis; and CaChA (**4**). ChA is of interest not only because it serves as a ligand in metal–organic compounds in plants [5,6,7], but also because of its various types of biological activity: it acts as an analgesic [8,9], an anti-inflammatory [9,10,11], an immunomodulator [12], an inhibitor of glutamate decarboxylase [13], an anti-cancer agent [14], and an inhibitor of histamine release from rat peritoneal mast cells [12] and reduces tumor necrosis factor-α (TNF-α) production [10]. Osteoprotective properties of some natural substances have been described [15,16,17], but the same properties of ChA and its derivatives have not been studied. Therefore, structural characterization of active substances isolated from SC extracts and their possible direct effect on osteogenic differentiation of multipotent mesenchymal stromal cells (MMSCs) promoting bone regeneration were of great interest.

## 2. Materials and Methods 

### 2.1. General Experimental Procedures

The NMR spectra for solutions of compounds in CD_3_OD, D_2_O and DMSO-d6 were recorded on the Bruker AV-600 spectrometer (600.30 (^1^H), 150.95 MHz (^13^C)) (Bruker BioSpin GmbH, Rheinstetten, Germany). Chemical shifts were reported in ppm (δ) relative to internal tetramethylsilane (TMS) for all the signs that could be identified with certainty. The melting points were determined on a Stuart SMF-38 melting point apparatus (Bibby Scientific, Staffordshire, UK) and are uncorrected. Ultraviolet (UV) spectra were obtained on an HP 8453 UV-Vis spectrometer (Hewlett-Packard, Germany) in EtOH solutions (10^−4^ mol/L). CHN analysis was carried out on a Carlo Erba 1106 elemental analyzer (Carlo Erba, Milan, Italy). Infrared spectra were obtained on a Nicolet 5700 (FT-IR, Thermo Fisher Scientific, Waltham, MA, USA) in tablets with potassium bromide. HR-MS spectra were recorded on a Thermo Scientific DFS (Thermo Fisher Scientific) mass spectrometer (evaporator temperature 200–220 °C, electronic ionization (EI) at 70 eV). X-ray structural study was performed on a Bruker KAPPA APEX II diffractometer (Bruker AXS, Karlsruhe, Germany) with a two-dimensional CCD detector (MoKα radiation with graphite monochromator, ω-φ-scanning). The inorganic components were studied by inductively coupled plasma mass spectrometry using Agilent 7900 JP 14,080,159 (Agilent Technologies, Tokyo, Japan) with decomposition of the organic matrix in the microwave system Speedwave MWS TM-3+ in the presence of nitric acid. HPLC was performed on a Shimadzu LC-20AD (Shimadzu Corporation, Kyoto, Japan) with Perfect Sil Target ODS-3 chromatographic column using a mixture of acetonitril-isopropanol (5:2 v/v) in a gradient of 0.1% trifluoroacetic acid.

### 2.2. Plant Material

Leaves of *S. controversa* were collected in the region of Irkutsk, Russia, during the flowering phase in July of 2013 and were air-dried. The plants were collected by Prof. A. A. Semenov and identified by Prof. M. N. Shurupova. A voucher specimen (No. TK-004605) has been deposited at the Herbarium of Tomsk State University (Tomsk, Russia).

### 2.3. Extraction and Isolation

Raw materials (600 g) were extracted with 40% ethanol (3 × 6000 mL, 80 °C, 1 h each). The extract was evaporated until it became an aqueous residue and then dried by convection. The dried extract (200 g) was dissolved in 1 L of water, resulting in a white amorphous precipitate (R1), which was separated and washed with water (**4**, 14 g, 70 mg/g of extract). The aqueous solution of the extract was treated sequentially in a separating funnel with CHCl_3_ (3 × 200 mL), ethyl acetate (6 × 200 mL), and n-butanol (10 × 200 mL). The resulting water residue was concentrated under vacuum to 0.5 L, and 2 L of 96% ethanol was added for the precipitation of polysaccharides. After the resulting precipitate was removed, the solution was concentrated under vacuum until the ethanol was completely removed. An amount of 1.5 L of acetone was added to the resulting aqueous solution (0.5) and kept for 24 h at a temperature of 18 °C until the white amorphous precipitate was precipitated (R2, 9 g). Through chromatography of R2 (0.2 g) on polyamide (1.2 × 25 cm; Woelm) using ethanol with a gradual increase in the content of water (5–50%), component **1** was isolated (yield 0.180 g, 45 mg/g of extract). Component **1** was dissolved in methanol by heating and adding concentrated HCl 3:1 (v/v). After cooling, **2** was obtained in the form of needle crystals. R1 was dissolved in the mixture of water and dilute HCl 5:1 *v/v* (350 mL) under heat (80 °C) and butanol (3 × 100 mL) was used to extract. A white powder (R3) was obtained by removing the butanol and drying. Through chromatography of R3 (0.9 g) on silica gel (1.2 × 65 cm; Lachema L 100/160) using CHCl_3_ with a gradual increase in the content of methanol (5–50%), components **3** (yield 0.55 g) and **1** (yield 0.35 g) were successively obtained.

### 2.4. 4-oxo-4H-pyran-2.6-dicarboxylic acid (1)

White amorphous powder (H_2_O); M.p. 265 °C; UV (H_2_O) nm: 272; IR (KBr, ν, cm^−1^): 3437 (OH), 3067 (CH_2_), 1702 (C=O), 1642 (-C=C-), 1405, 1361(OH), 1123 (C-O), 960, 914, 883, 812, 796, 730, 618, 539, 465; ^1^H-NMR (600 MHz, D_2_O) δ: 7.02 (2H, s); ^13^C-NMR (150 MHz, DMSO-d6) δ: 180.0 (CO), 152.9, 118.2 (C=C), 160.2 (COOH); MS *m/z:* 184 (M^+^), 139, 69; A*nal*. calc. for C_7_H_4_O_6_ (184.07): C 45.67, H 2.19; found: C 45.65, H 2.17. 

### 2.5. 4-oxo-4H-pyran-2.6-dicarboxylic acid methyl ester (2)

White crystalline powder (MeOH); M.p. 186–187 °C; *Crystallographic data:* C_8_H_6_O_6_ *M* 198.13, monoclinic, *P2_1_/c*, *a* 8.1967(5), *b* 11.8401(6), *c* 8.8851(5) Å, β 95.980(3)°, *V* 857.60(8) Å^3^, *Z* 4, *D*_calcd_ 1.535 g·cm^−3^, *μ*(Mo-*K*α) 0.136 mm^−1^, F(000) 408, (θ 2.50 – 30.36°, completeness 99.6%), T 296(2) K, colorless, (0.75 × 0.50 × 0.50) mm^3^, transmission 0.8293 – 0.8622, 15,916 measured, 2584 independent (*R*_int_ 0.0215), 131 parameters, *R*_1_ 0.0534 (for 1991 observed *I>* 2*σ*(*I*)), *wR*_2_ = 0.1846 (all data), goodness of fit 1.112, largest differential peak and hole 0.390 and −0.231 e.A^−3^. Reflection intensities were corrected for absorption by the SADABS program [18]. The structure was solved by direct methods using the SHELXS-97 program [19] and refined by the anisotropic (isotropic for all H atoms) full-matrix least-squares method against *F^2^* of all reflections by SHELX-97 [19]. The hydrogen positions were calculated geometrically and refined in a riding model, except for the hydrogen positions in the hydroxyl group, which were refined freely. Crystallographic data for the structure have been deposited at the Cambridge Crystallographic Data Centre as supplementary publication no. CCDC 1879739. Copies of the data can be obtained free of charge on application to CCDC, 12 Union Road, Cambridge CB21EZ, UK (fax: +44 122 3,336,033 or e-mail: deposit@ccdc.cam.ac.uk; internet: www.ccdc.cam.ac.uk). 

### 2.6. 4-oxo-4H-pyran-2.6-dicarboxylic acid n-monobutyl ester (3)

White crystalline powder (H_2_O); M.p. 170-171 °C; UV (EtOH) nm: 272; IR (KBr, ν, cm^−1^): 3461 (OH), 3105, 3066 (CH_2_), 2959, 2939, 2903, 2875 (CH_3_), 2567, 2457, 1748 (C=O), 1641, 1583 (-C=C-), 1477, 1417, 1393, 1360, 1277, 1247 (OH), 1136, 1118, 1062, 1032 (C-O), 965, 913, 885, 784, 740, 692, 532, 483, 460, 430; ^1^H-NMR (600 MHz, CD_3_OD) δ: 7.13 (m, 1H), 4.42 (t, 2H), 1.78 (m, 2H), 1.52 (m, 2H), 1.02 (t, 3H); ^13^C-NMR (150 MHz, CD_3_OD) δ: 181.7 (CO), 120.1 (C=C), 155.5, 154.8 (C=C), 161.4 (COOR), 160.4 (COOH), 67.8, 31.2, 19.8 (CH2), 13.9 (CH3); MS *m/z:* 240.0634 (M^+^), 185, 139, 69; A*nal*. calc. for C_11_H_12_O_6_ (240.06): C 55.04, H 5.00; found: C 55.86, H 4.87. 

### 2.7. [Ca(ChA)(H_2_O)_3_] (4)

White amorphous powder (H_2_O); calcium content 0.145 g/g; UV (EtOH) nm: 274; IR (KBr, ν, cm^−1^): 3516 (H_2_O), 3273, 3073, 2827 (CH_2_), 1639, 1617, 1597 (-C=C-), 1410, 1357, 1315, 1134, 1122, 973, 956, 921, 906, 806, 744, 723, 623, 548, 465; A*nal*. calc. for C_7_H_8_O_9_Ca (276.15): C 30.43, H 2.90, Ca 14.49; found: C 31.49, H 2.93, Ca 14.50.

### 2.8. Cell Culturing and In Vitro Staining

Adult human adipose-derived multipotent mesenchymal stromal cells (hAMMSCs) were isolated from lipoaspirates of healthy volunteers (permission no. 7 from 9 December 2013; the Local Ethics Committee, Innovation Park, Immanuel Kant Baltic Federal University) as described in [20]. The cells were stained using a Phenotyping Kit, human (130-095-198) and Viability Fixable Dyes (Miltenyi Biotec, Bergisch Gladbach, Germany), and the results were analyzed with a MACS Quant flow cytometer (Miltenyi Biotec, Bergisch-Gladbach, Germany) and KALUZA Analysis Software (Beckman Coulter, Brea, CA, USA) in accordance with the manufacturer’s instructions. More than 98% of the viable cells expressed CD73, CD90, or CD105 markers and did not display CD45, CD34, CD20, or CD14 markers (less than 2%).

To confirm the morphofunctional nature of hAMMSCs, 5 × 10^4^ viable cells/mL were cultivated in 1.5 mL of medium with reagent from a StemPro® Differentiation Kit (Thermo Fisher Scientific) for 21 days, and the medium was exchanged every 3–4 days. After removal of the supernatants, the adherent cells were dried, fixed, and stained with the dyes. The AMMSC culture was estimated by cell staining with alcian blue (Sigma-Aldrich, St. Louis, MO, USA), which enabled visualization of proteoglycan synthesis by chondrocytes; alizarin red S (Sigma-Aldrich), which identified mineralization of the intercellular matrix by osteoblasts; and oil red (Sigma-Aldrich), which detected neutral triglycerides and lipids in adipocytes. All stainings were performed as recommended by the manufacturer. The results were assessed with a Zeiss Axio Observer A1 microscope (Carl Zeiss Microscopy, LLC, Thornwood, NY, USA) using ZEN 2012 software (Carl Zeiss Microscopy, LLC). The cell culture showed a presence of osteoblasts, chondrocytes, and adipocytes. This confirmed that AMMSCs belonged to the pool of stem cells according to the recommendations of the International Society for Cellular Therapy (ISCT) [21,22]. 

To determine the effect of substances **1**, **3**, and **4** on osteogenic (into osteoblasts) differentiation of hAMMSCs, cells were cultured in an incubator (Sanyo, Osaka, Japan) at 100% humidity with 5% carbon dioxide at 37 °C for 21 days (the medium was replaced with fresh medium every 3–4 days). The cell suspension was prepared at an initial concentration of 5 × 10^4^ viable cells/mL in 1.5 mL of the following culture medium: 90% αMEM medium (Gibco Life Technologies; Grand Island, NY, USA), 10% fetal bovine serum (Sigma-Aldrich), 50 mg/L of gentamicin (Invitrogen, Carlsbad, CA, USA) and sterile L-glutamine solution freshly added to a final concentration of 280 mg/L (Sigma-Aldrich). The compounds (final concentration 10, 30, or 50 mg/L) were added to the hAMMSC culture in plastic wells of a 24-well flat-bottom plate (Orange Scientific, Braine-l’Alleud, Belgium) initially and each time the medium was replaced.

To establish the self-differentiation potency of hAMMSCs caused by tested compounds, the culture medium had no osteogenic supplements (β-glycerophosphate, dexamethasone, and ascorbic acid). The wells were air dried. The attached cells in the 21-day culture were fixed with 10% formalin for 1 h, washed with phosphate buffer and stained with 2% alizarin red (Sigma-Aldrich) to visualize the extracellular matrix. The osteoblasts with mineralization sites around them were stained in cell cultures with 2% alizarin red S as described above. Digital images of the hAMMSC culture were obtained with a microscope as described above. The average areas of alizarin red staining (in mm^2^) were calculated via quantitative computer histomorphometry with the help of ImageJ v. 1.43 software (http://www.rsb.info.nih.gov/ij) and total areas (average areas × numbers of stained sites) were determined in each well.

The other three wells for each dose of tested substances were used to evaluate their possible cytotoxicity in 21 days of hAMMSC cultivation. The cells were disaggregated with 0.05% trypsin (PanEco, Moscow, Russia) in 0.53 mM EDTA (Sigma-Aldrich), and the concentration of viable cells was determined with a Countess^TM^ Automated Cell Counter (Invitrogen) using 0.4% trypan blue solution (Invitrogen).

### 2.9. Statistical Analysis 

Statistical processing of results was carried out using the SPSS 17.0 statistical analysis software package. The following distribution parameters were calculated: the mean (X) and standard deviation (SD) or the median (Me), 25% (Q1) and 75% (Q3) quartiles. The normality of distribution was defined by Kolmogorov-Smirnov test. Because of non-normal distribution, non-parametric Mann-Whitney *U* test was used to evaluate the significant differences between the samples. Statistically significant differences were considered at a significance level of *p* < 0.05. The relationship between the studied parameters was established via regression analyses. The coefficients (r) were kept at a significance level greater than 95%.

## 3. Results

### 3.1. Isolation of Components and Determination of Their Structure

**Figure 1:**
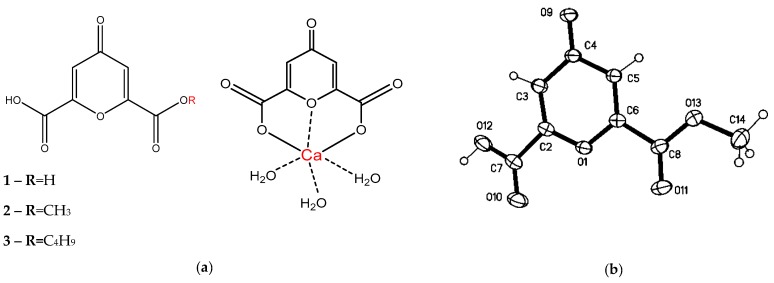
The structure of compounds **1–4** (**a**) and XRD structure of **2** (thermal ellipsoids are drawn at the 30% probability level) (**b**).

The subject of this study was the water-soluble fraction of extract from *SC* leaves. Dried extract from these leaves was dissolved in water, resulting in precipitation of a white amorphous sediment (R1). The aqueous solution of the extract was pretreated with lipophilic solvents (chloroform and ethyl acetate) to purify the hydrophilic components, and then n-butanol was used to isolate flavonoids from the purified extract. The structure and activity of these flavonoids have been described earlier [4]. The remaining hydrophilic fraction constitutes about 85% of the extract. Since the similar properties of components in this fraction complicated its separation, an optimal scheme was determined for separating the fraction’s major components. The resulting aqueous residue was concentrated under vacuum and polysaccharides were precipitated by adding (1:4 v/v) of 96% ethanol. Then acetone was added to the aqueous residue, which produced an amorphous precipitate (R2). During elution of R2 using a mixture of ethanol and water (1:1 v/v) on polyamide, compound **1** was obtained in the form of a white amorphous powder, identified as a single peak with t_r_ 3.4. CHN analysis showed the molecular content of this compound to include 45.65% carbon and 2.17% hydrogen. One peak corresponding to the aromatic proton was detected in the NMR spectrum (δ_H_ 7.15 s). Signals characterizing the symmetry of the molecule were detected (δ_C_ 180.0 (C=O); 152.9, 118.2 (-C=C-); 160.2 (-COOH)). Mass Spectrometry was used to determine the molecular cation radical *m/z:* 184.0006 (M^+^, C_7_H_4_O_6_^+^; calc.184.0002) and main fragmentation ions *m/z:* 139 (−45, acetyl); 69 (-70, pyrrolidone) (Figure S1). Based on the data obtained, it can be assumed that **1** is ChA. Recrystallization of ChA was accomplished by dissolving it in methanol under heat and adding concentrated HCl 3:1 (v/v). After cooling, the substance crystallized in the form of needle crystals (**2**). The structure of **2** was determined to be 4-oxo-4*H*-pyran-2.6-dicarboxylic acid monomethyl ester by using single crystal X-ray analysis (Figure 1b), which confirmed the structure of ChA isolated from *SC*.

The purified R1 is metal–organic compound **4**. By using ICP-MS on **4**, we found 0.145 g/g of calcium as an inorganic component. To study the organic component, we dissolved **4** in water mixed with dilute hydrochloric acid (5:1 v/v) under heat (80 °C) and used n-butanol to extract the organic component. Evaporation of butanol fraction yielded an ethanol-soluble white powder (R3) with two peaks in HPLC with t_r_ 3.5 and 25.4 (**3**) (Figure S2). By using chloroform elution with a gradual increase of the methanol gradient to separate R3 on silica gel, we obtained two components in a weight ratio of 1:1.6. The substance with t_r_ 3.5 has NMR data similar to the previously isolated ChA. The NMR spectra for **3** have peaks corresponding to the aromatic proton (δ_H_ 7.13 m), oxomethylene protons (δ_H_ 4.42 t), methylene protons (n_H_ 1.78 m, 1.52 m), and methyl groups (δ_H_ 1.02 t) (Figure S3). The NMR data (δ_C_ 181.7 (CO), 120.1 (C=C), 155.5, 154.8 (C=C), 160.4 (COOH), 161.4 (COOR), 67.8, 31.2, 19.8 (CH2), 13.9 (CH3)) show the asymmetry of the molecule (Figure S4). MS was used on **3** to determine the molecular cation radical *m/z:* 240.0634 (M^+^, C_11_H_12_O_6_^+^; calc. 240.0628) and main fragmentation ions *m/z*: 185, 139, 69 (Figure S5). Based on spectral characteristics, substance **3** is the n-monobutyl ester of ChA (BuChA). It is probable that BuChA formed at the stage of R1 dissolution and subsequent extraction with n-butanol in an acidic medium. Infrared spectrum was used on **4** to detect the absorption band at 3516 (H_2_O); 3273 (OH); 3073, 2827 (CH_2_); 1639, 1617, 1597 (-C=C-); 1410, 1357, 1315 (OH); 1134 (C-O) cm^−1^. The absorption band characteristic of the coordinated water molecule is present in the spectrum and correlates well with the band described for a similar compound [6]. In agreement with involvement of the –COOH group in the formation of chemical bonds, no absorption bands were observed for the complex at 1702 cm^−1^, compared to the free ligand. For comparison, the IR spectra of ChA, BuChA, and CaChA are provided in Figure S6 and Figure S7. The number of water molecules was determined by comparing the value calculated for C_7_H_8_O_9_Ca: C 30.43, H 2.90, Ca 14.49%, and the value obtained for it in the experiment: C 31.49, H 2.93, Ca 14.50% (Figure 1a).

### 3.2. Study of Osteogenic Differentiation of hAMMSCs In Vitro

**Figure 2:**
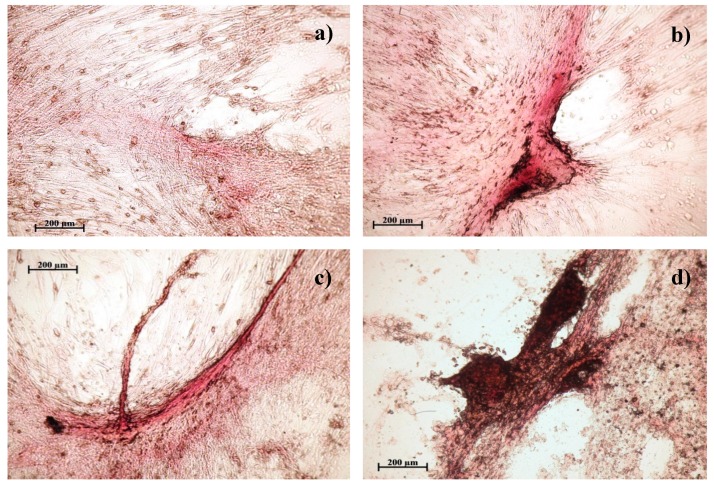
Culture of human multipotent mesenchymal stem cells after 21 days of cultivation in a standard nutrient medium: a—control; b—with the addition of 50 mg/L of chelidonic acid; c—with the addition of 50 mg/L of n-monobutyl ester of chelidonic acid; d—with the addition of 50 mg/L of chelidonic acid complex with calcium. Staining with alizarin red.

When hAMMSCs were cultivated in a standard nutrient medium without the addition of compounds, the main part of the well was occupied by fibroblast-like cells weakly stained by alizarin red (Figure 2). A small part of the poorly colored cells had an irregular, polygonal shape. Such morphology is typical for hAMMSCs during prolonged cultivation. Formation of intensely colored fibrous-like structures was not observed in the cell monolayer.

In the groups cultured in the standard medium with the addition of the studied compounds, the hAMMSCs did exhibit a change in morphology, depending on the concentration of the introduced samples. For all these samples, an increase in the intensity of red color and the number of stained sites indicates a differentiation of hAMMSCs into osteoblasts of deposed calcium salts in the intercellular matrix [23]. Therefore, when a small concentration of sample (10 mg/L) was added, it was noted that the cells changed shape by decreasing in length and the number of outgrowths. Single foci of calcification appeared in the intercellular matrix, which was intensely stained with alizarin red, which indicates the osteogenic differentiation of individual stem cells. When the concentration of the introduced sample (30 and 50 mg/L) increased, individual foci of mineralization as well as extended, intensely colored multilayer fibrous-like structures were observed. The formation of such strands is a result of the merger of individual areas of mineralization and indicates a significant increase in osteoblastic differentiation in the culture of hAMMSCs under the influence of the studied compounds.

The results of the in vitro study showed that a dose-dependent nonlinear reduction in the number of viable stem cells was observed after long-term cultivation of hAMMSCs in a standard nutrient medium in the presence of ChA (Table 1). At the same time, ChA at a dose of 10 mg/L contributes to the survival of AMMSCs, but not to their differentiation into osteoblasts, since the area of coloring with alizarin red at the sites of mineralization of the intercellular matrix (function of osteoblasts) [23] is less than in the control (Table 1, Figure 2). The total area of calcification sites increases linearly with increasing doses of ChA up to 30–50 mg/L. However, the cell culture survival rate significantly dropped (Table 1 and Table 2).

Similar BuChA inhibiting influence on cell culture survival was found at a dose of 50 mg/L. Moreover, it decreased hAMMSC differentiation into osteoblasts at a dose of 30 and 50 mg/L as compared with corresponding doses of ChA (Table 1 and Table 2).

In the presence of CaChA, the hAMMSC culture increased (up to 156% of the control at a dose of 50 mg/L) the number of viable cells in nonlinear dose dependence (Table 1) with simultaneous stimulation (3–6 times) of their differentiation into osteoblasts (as compared with control and corresponding doses of other substances), which was identified by increasing the total areas of alizarin red staining (Table 2, Figure 2).

It should be noted the average areas of the sites stained with alizarin red did not differ statistically from the control level (Table 2). There was mainly increased number of stained sites in cell culture followed by corresponding growth of total areas of staining.

## 4. Discussion

Thus, ChA in the native state and in the complex with calcium [Ca(ChA)(H_2_O)_3_], named saucalchelin, was isolated from the extract of *Saussurea controversa* leaves with yield of 45 mg/g and 70 mg/g of extract, respectively. It should be noted that derivatives of γ-pyron in the genus *Saussurea* DC and *Asteraceae* family had not been described previously. The metal–organic compound CaChA isolated from *SC*, represents the complex of ChA with calcium and the associated three water molecules (Figure 1a). It is known that calcium complexes form stable hydrates in an aqueous medium which can be extracted with organic solvents only when the coordination water is replaced with a polar organic solvent, in our case, n-butanol in an acidic medium. Calcium can exhibit coordination numbers 6 or 8 in the formation of complexes with organic molecules. The ability of calcium ions to form complex compounds with different structures allows them to easily adapt to the surrounding donor atoms of bioligands and serve as bridges between ligands, which is observed in CaChA.

*SC* can be considered as a concentrator of ChA both in native form and as a metal–organic compound with calcium. ChA and CaChA were isolated from *SC* extract, which showed osteogenic activity in early experiments in vivo [4]. This activity may be summarized from direct and indirect (through immunoneuroendocrine network) effects of extracts and their compounds on bones. Therefore, the study of direct in vitro action of substances on MMSCs was of interest. Also, the aim was to study the effect of ChA esterification on the expected activity.

In vitro staining of AMMSC culture with alizarin red is morphological criterion for defining stromal cell differentiation into osteoblasts [21,22]. According to our results of in vitro stromal cell testing (Table 1 and Table 2), ChA and CaChA proved to be weak activators of osteogenic differentiation of the AMMSC culture because only certain their doses caused cell culture staining with alizarin red. For all this, high doses of ChA and CaChA showed side effects on AMMSCs, which led to inhibition of the number of cultured cells that is dependent on a well-known balance of the processes of cell proliferation, differentiation and death. In turn, investigated doses of CaChA significantly stimulated the growth of cell viability (Table 1) and mineralization of the hAMMSC culture (Table 2) compared to both the control and other substances.

Local mineralization of studied in vitro hAMMSC culture was known earlier [24] and it corresponds to focal bone tissue regeneration in vivo [23]. In this connection, an increasing number of the sites of non-uniform staining with alizarin red counted in favour of enhanced local differentiation of AMMSCs into osteoblasts caused by tested substances. Vice versa, only diffuse weak staining could be detected in case of simple deposition of the calcium containing CaChA on the cells. Moreover, there was also focal alizarin red staining in an absence of calcium in the structure of substances (Figure 1, Table 2).

Thus, the osteogenic effect of the tested substances on hAMMSCs increases as follows: BuChA (10 mg/L, only) ~ ChA (30 and 50 mg/L) < CaChA (10–50 mg/L). CaChA may be a promising substance for accelerating bone tissue regeneration and engineering. It is interesting to propose the pathways for CaChA osteogenic activity. Of course, well-known signaling pathways (bone morphogenetic protein 2 (BMP-2); runt-related transcription factor 2 (RUNX2), Wnt) for our substance could be potential targets [25] that need further investigation. Besides, we believe that small molecules of Ca-containing CaChA may influence similarly to osteogenic small molecules such as β-glycerophosphate, dexamethasone, and ascorbic acid; adenosine (*via* phosphate–adenosine triphosphate (ATP)—adenosine A2b receptor (A2bR) axis) and helioxanthin derivative 4-(4-methoxyphenyl)-pyrido [40.30:4.5]thieno-[2,3-b]pyridine-2-carboxamide [26], or calcium (through Ca^2+^-sensing receptor), especially [27].

Two types of CaR agonists are known. Type I agonists (calcium and other divalent (Mg^2+^) and polyvalent cations including gadolinium, neomycin, polyamines), independently activate the receptor that stimulates osteoblasts differentiation and mineralized nodule formation. In contrast, Type II agonists are positive allosteric modifiers that potentiate the response to type I ligands. These include aromatic amino acids and the calcimimetic drugs [27].

There is an evidence of the participation of extracellular adenosine (ATP-metabolized) and A2bR in mineralized matrix-mediated osteogenic differentiation of human MMSCs [28], the secretion of adenosine by MMSCs, and the influence of extracellular adenosine in promoting osteoblast functions [29]. Thus, autocrine/paracrine stimulation of MMSC osteogenic differentiation by small molecules may occur.

## 5. Conclusions

Chelidonic acid and its complex with calcium [Ca(ChA)(H_2_O)_3_], named saucalchelin, was isolated for the first time for the *Asteraceae* family from *Saussurea controversa* leaves. These compounds have a high content in the extract and probably provide osteogenic activity of *Saussurea controversa*. Chelidonic acid and its n-butyl ester caused in vitro both weak osteogenic differentiation of hAMMSCs and cytotoxicity at a dose of 50 mg/L. At the same time, the complex compound of ChA with calcium, saucalchelin, strongly stimulated growth of cell biomass and morphological signs of hAMMSC differentiation into osteoblasts.

## Figures and Tables

**Table 1:** The indices of human adipose-derived multipotent mesenchymal stromal cell (hAMMSC) viability after 21 days of culturing in the presence of tested compounds, X ± standard deviation (SD).

Group, *n* = 3		Dose mg/L	Number of viable cells, ×10^4^ cells/mL, n_1_ = 6	*U* Criterion	Equation of dose-dependent linear regression
1	*Control	0	9.5 ± 0.61		-
2	Cells + ChA	10	21.7 ± 3.96	U_1_<0.05	Y = 24.0833–0.405X; P = 0.2180
		30	8.6 ± 1.73		
		50	5.5 ± 1.08	U_1_<0.05 U_2_<0.05	
3	Cells + BuChA	10	8.7 ± 0.23		Y = 10.4000–0.100X; P = 0.3469
		30	8.8 ± 0.24		
		50	4.7 ± 0.13	U_1_<0.05 U_2_<0.05	
4	Cells + CaChA	10	11.3 ± 1.01	U_1_<0.05	Y = 10.1083 + 0.0875X; P = 0.1934
		30	12.1 ± 1.07	U_1_<0.05	
		50	14.8 ± 1.26	U_1_<0.05 U_2_<0.05	

Statistical significances are shown according to the Mann-Whitney *U* test: U_1_ – with the control; U_2_ – with the doses of 10 and 30 mg/L; n – the number of tested samples for each dose; n_1_ – the number of duplicate probes for each dose. *Control— Stem cells cultured on plastic surface without compounds.

**Table 2:** In vitro effect of the compounds on hAMMSC osteogenic differentiation with mineralization of cell culture matrix, X ± SD, Me(Q1–Q3).

Group, *n* = 3		Dose, mg/L	Total area of the sites of cell culture mineralization, mm^2^, *n* = 3	*U* criterion, statistical significance	An average area of the mineralization sites, mm^2^	*U* criterion, statistical significance	Equation of dose-dependent linear regression
1	*Control	0	1.166 ± 0.433	-	0.027(0.013–0.095) n_1_ = 14	-	-
2	Cells + ChA	10	0.589 ± 0.262	-	0.033(0.009–0.053) n_1_ = 13	-	Y = 0.098X–0.379; P = 0.0064
		30	2.582 ± 0.470	U_1_ <0.05	0.043(0.018–0.121) n_1_ = 31	-	
		50	4.507 ± 0.097	U_1_<0.05 U_2_<0.05	0.060(0.024–0.096) n_1_ = 56	-	
3	Cells + BuChA	10	1.904 ± 0.306	U_1_ <0.05 U_3_ <0.05	0.021(0.009–0.133) n_1_ = 25	-	Y = 0.008X + 1.686 P = 0.6263
		30	1.649 ± 0.563	U_3_ <0.05	0.026(0.010–0.077) n_1_ = 32	-	
		50	2.222 ± 0.599	U_1_ <0.05 U_3_ <0.05	0.042(0.013–0.123) n_1_ = 27	-	
4	Cells + CaChA	10	3.266 ± 0.207	U_1_ <0.05 U_3_ <0.05	0.036(0.021–0.069) n_1_ = 60	-	Y = 0.069X + 3.228 P = 0.4365
		30	6.620 ± 0.117	U_1_ <0.05 U_3_ <0.05	0.057(0.012–0.187) n_1_ = 58	U_2_<0.05	
		50	6.041 ± 0.033	U_1_ <0.05 U_2_<0.05 U_3_ <0.05	0.049(0.027–0.124) N _1_= 70	U_2_<0.05	

Statistical significances are shown according to the Mann-Whitney *U* test: U_1_ – with the control; U_2_ – with the doses of 30 and/or 10 mg/l; U_3_ – with the corresponding doses of other substances; n – the number of tested wells of cultural plate for each dose; n_1_ – the number of mineralization sites summarized in three wells for each dose. In each well the areas of mineralization sites were determined as well both total and average values for each dose were calculated. *Control—Stem cells cultured on plastic surface without compounds.

## Supplementary Materials

Figures S1–S7 are available online at https://www.mdpi.com/2218-273X/9/5/189/s1.

## Sample Availability

Samples of the compounds 1–4 are available from the authors.
