# Supplementary material for: Chelidonic Acid and Its Derivatives from Saussurea Controversa: Isolation, Structural Elucidation and Influence on the Osteogenic Differentiation of Multipotent Mesenchymal Stromal Cells In Vitro
Source: Biomolecules. 2019 May 16;9(5):189. doi: 10.3390/biom9050189 (PMC6572306; doi:10.3390/biom9050189)
Supplement: Supplementary file 1 [file biomolecules-09-00189-s001.pdf]

Supplementary

Chelidonic Acid and its Derivatives from *Saussurea Controversa*: Isolation, Structural elucidation and Influence on the Osteogenic Differentiation of Multipotent Mesenchymal Stromal Cells in Vitro

Elena Avdeeva [1*], Elvira Shults [2,4], Tatyana Rybalova [3,4], Yaroslav Reshetov [1], Ekaterina Porokhova[5], Irina Sukhodolo [5], Larisa Litvinova [6], Valeria Shupletsova [6], Olga Khaziakhmatova [6], Igor Khlusov [5,7], Artem Guryev [1] and Mikhail Belousov [1,7]

1 Department of Pharmaceutical Analysis, Siberian State Medical University, Tomsk, 634050 ,Russia; ferroplex2013@yandex.ru (Y.R.); titan-m@mail.ru (A.G.); mvb63@mail.ru (M.B)
2 Laboratory of Medicinal Chemistry, Novosibirsk Institute of Organic Chemistry, Siberian Branch, Novosibirsk, 630090, Russia; schultz@nioch.nsc.ru (E.S.)
3 Center of Spectral Investigations, Novosibirsk Institute of Organic Chemistry, Siberian Branch, Novosibirsk, 630090, Russia; rybalova@nioch.nsc.ru (T.R)
4 Novosibirsk State University, 2 Pirogova St., Novosibirsk 630090, Russia
5 Department of Morphology and General Pathology, Siberian State Medical University, Tomsk, 634050 ,Russia; porohova_e@mail.ru (E.P.); staranie@mail.ru (I.S.); khlusov63@mail.ru (I.K.)
6 Basic Laboratory of Immunology and Cell Biotechnology, Immanuel Kant Baltic Federal University, Kaliningrad, 236041, Russia; larisalitvinova@yandex.ru (L.L.); vshupletsova@mail.ru (V.S.); hazik36@mail.ru (O.K.)
7 Research School of Chemistry & Applied Biomedical Sciences, Tomsk Polytechnic University, Tomsk, 634050, Russia
* Correspondence: elenaavdeev@yandex.ru; Tel.: +7-983-344-7381 (E.A.)

TABLE OF CONTENTS

**Figure 1S.** MS-spectrum of chelidonic acid (**1**)	**S2**
**Figure 2S.**The chromatographic profile of chelidonic acid, **1** (A) and R3 (B)	**S3**
**Figure 3S.** ^{1}H NMR (CD_3OD, 600 MHz) of n-monobutyl ester of chelidonic acid (**3**)	**S4**
**Figure 4S.** ^{13}C NMR (CD_3OD, 150 MHz) of n-monobutyl ester of chelidonic acid (**3**)	**S5**
**Figure 5S.** MS-spectrum of n-monobutyl ester of chelidonic acid (**3**)	**S6**
**Figure 6S.** IR-spectrum (KBr, cm^{-1}) of n-monobutyl ester of chelidonic acid (**3**)	**S7**
**Figure 7S.** IR-spectrum (KBr, cm^{-1}) of chelidonic acid (A) and [Ca(ChA)(H_2O)$_3$] (B)	**S8**

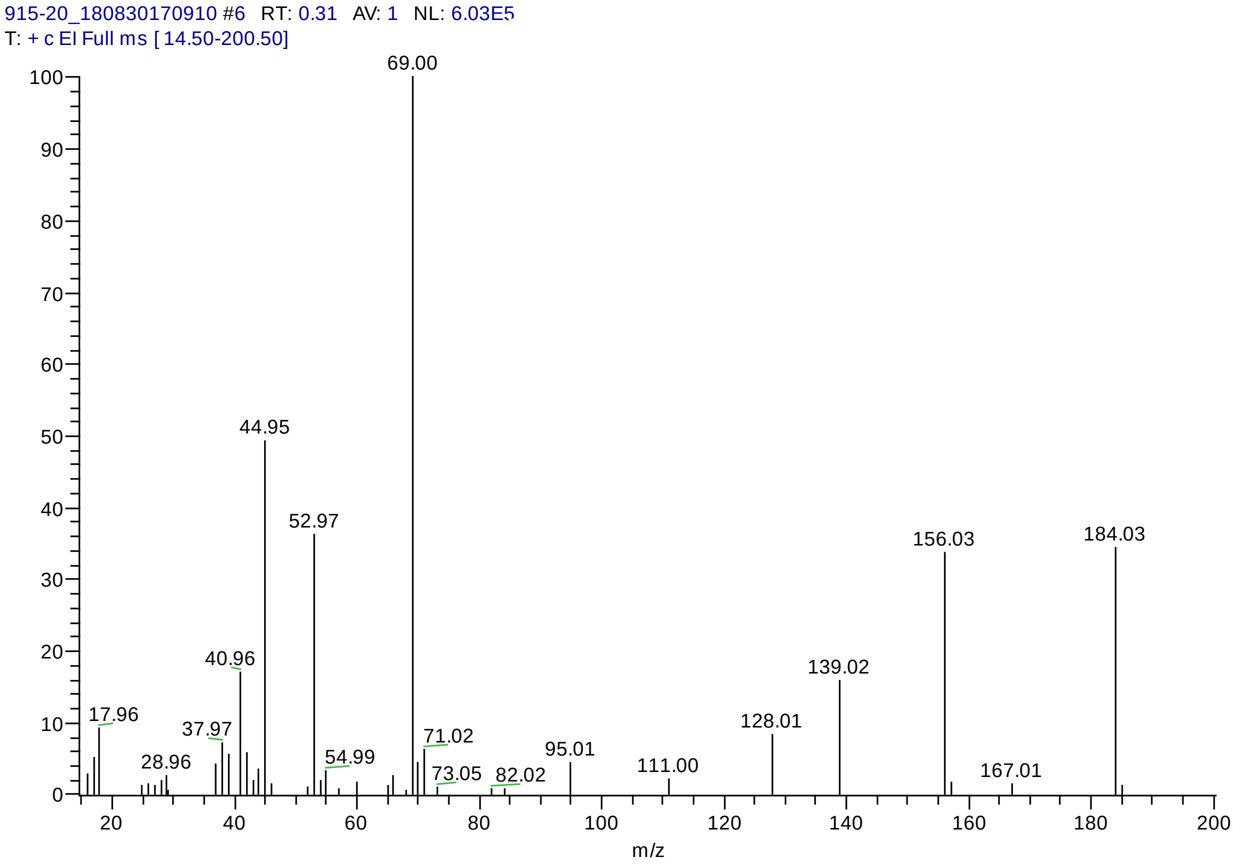

Figure 1S. MS-spectrum of chelidonic acid (**1**)

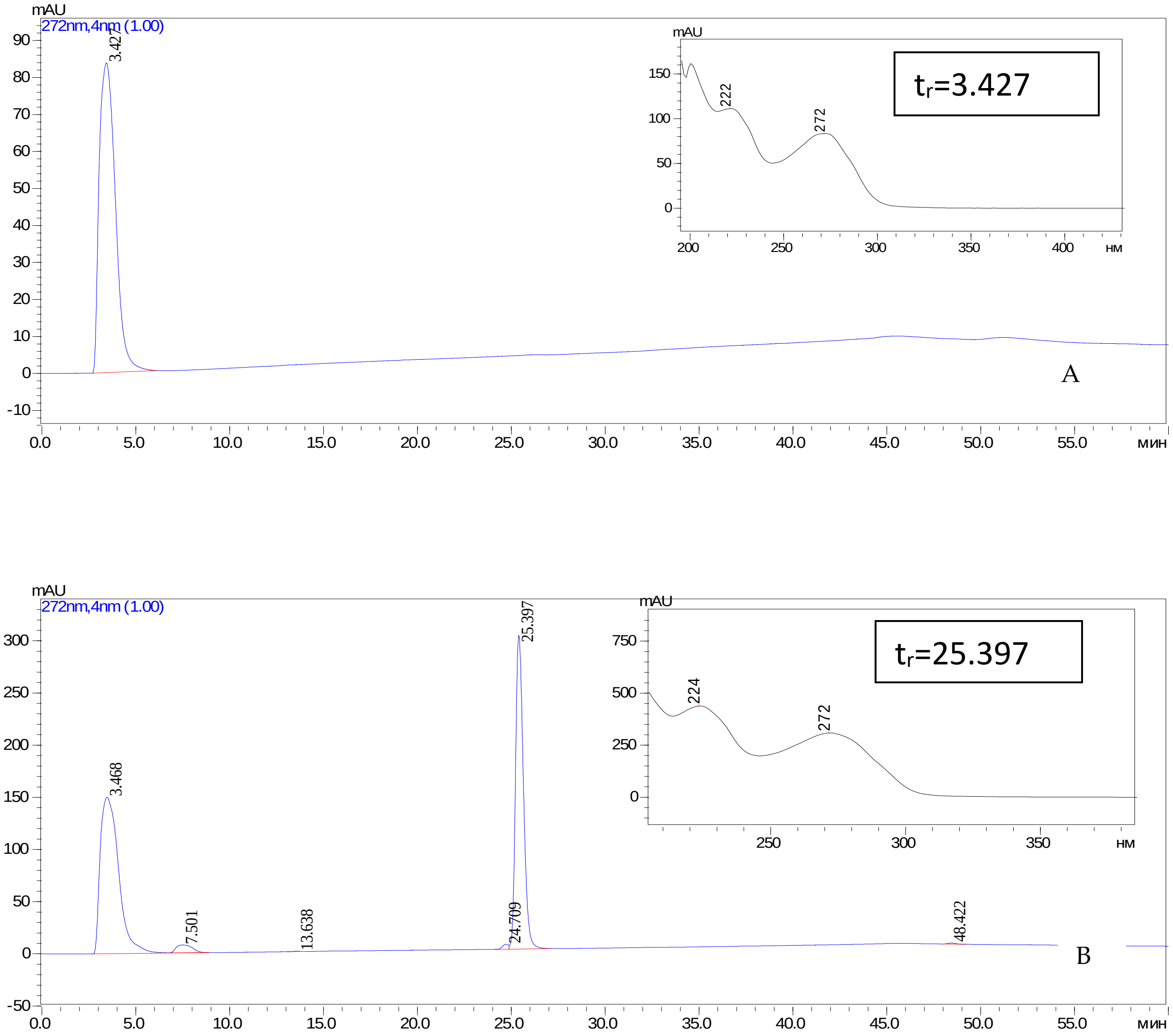

Figure 2S. The chromatographic profile of chelidonic acid, **1** (A) and R3 (B).

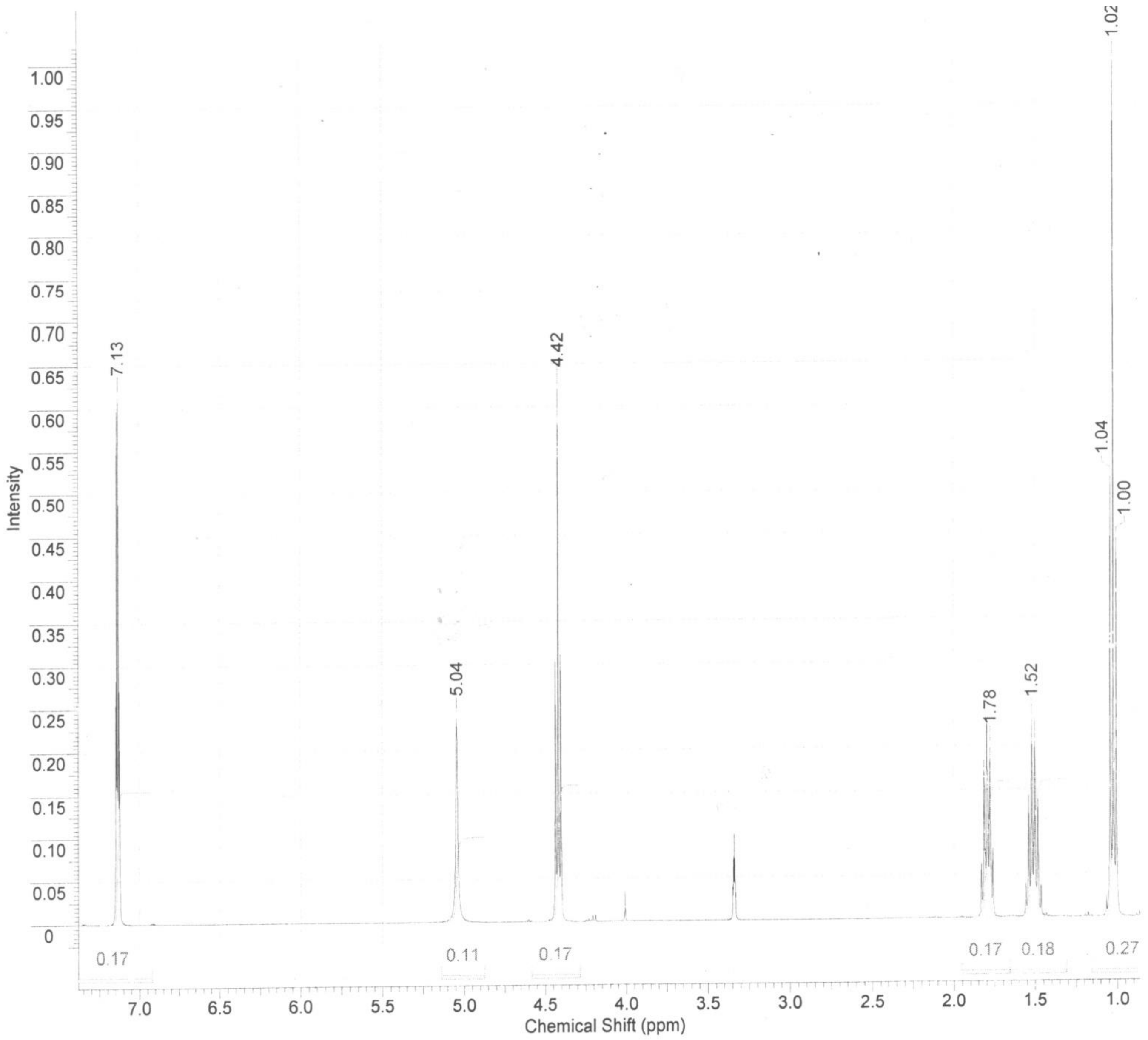

Figure 3S. ^{1}H NMR (CD_3OD, 600 MHz) of n-monobutyl ester of chelidonic acid (**3)**

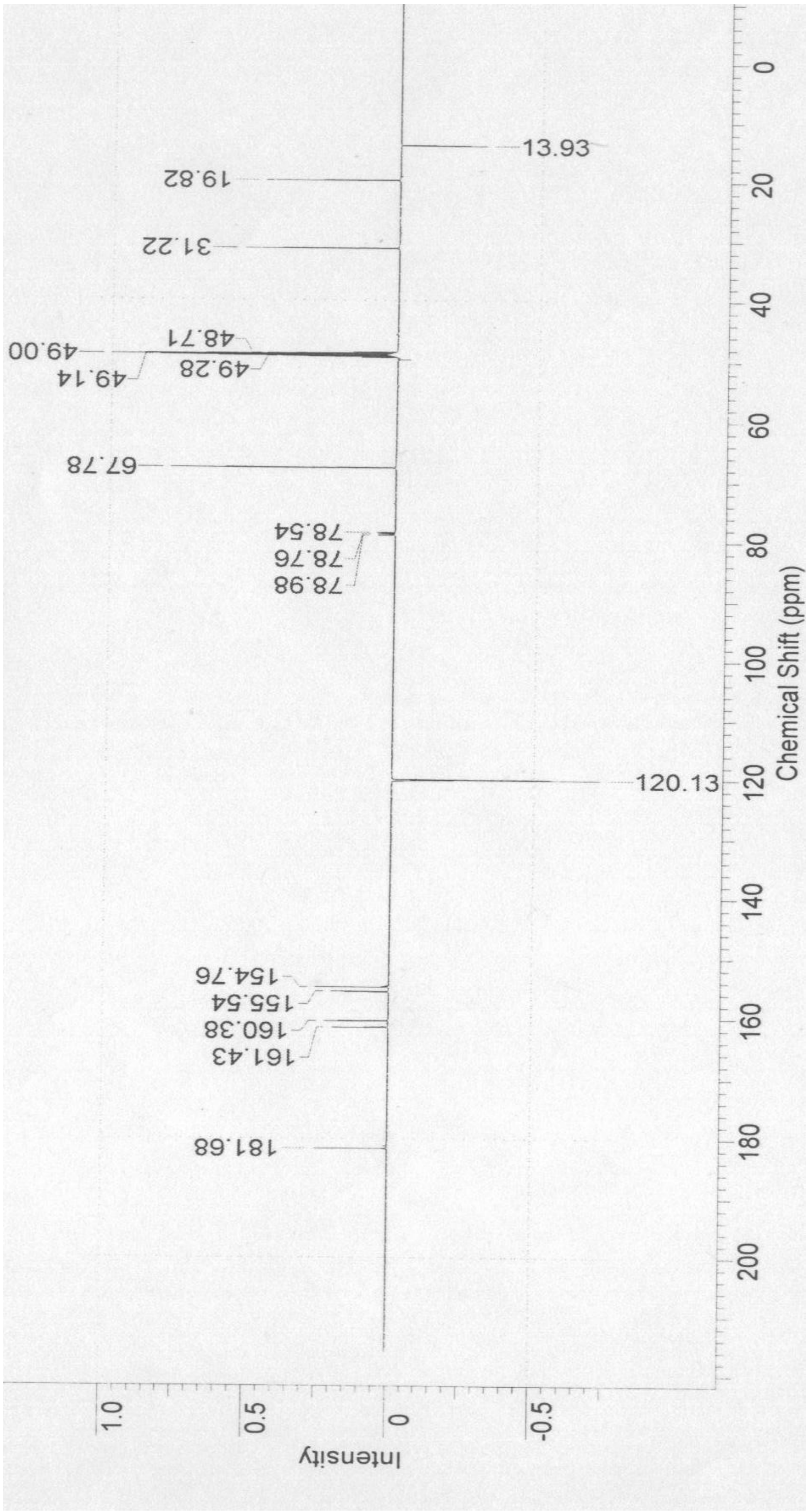
13.93
19.82
31.22
48.71
49.00
49.14
49.28
67.78
78.54
78.76
78.98
120.13
154.76
155.54
160.38
161.43
181.68
Chemical Shift (ppm)
Intensity

Figure 4S. ^{13}C NMR (CD_3OD, 150 MHz) of n-monobutyl ester of chelidonic acid (**3**)

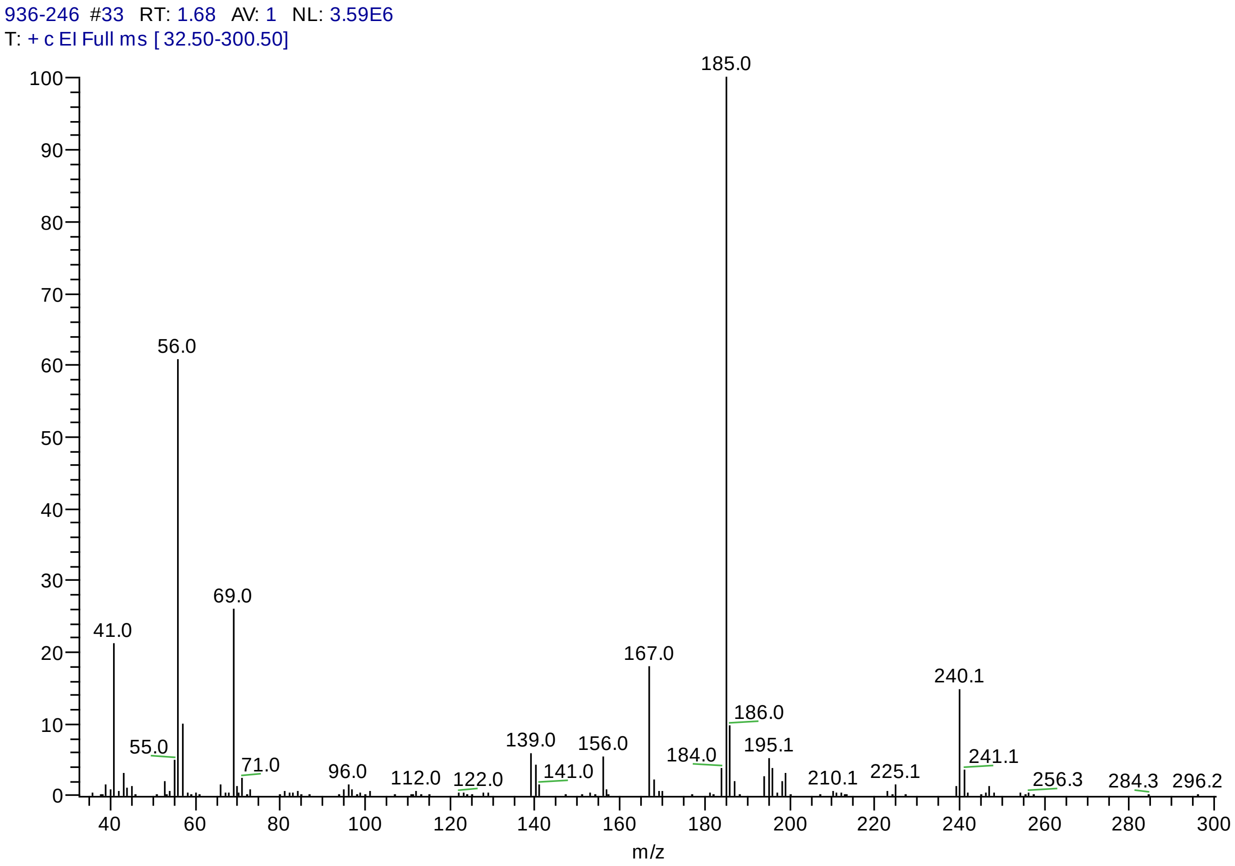

Figure 5S. MS-spectrum of n-monobutyl ester of chelidonic acid (**3**)

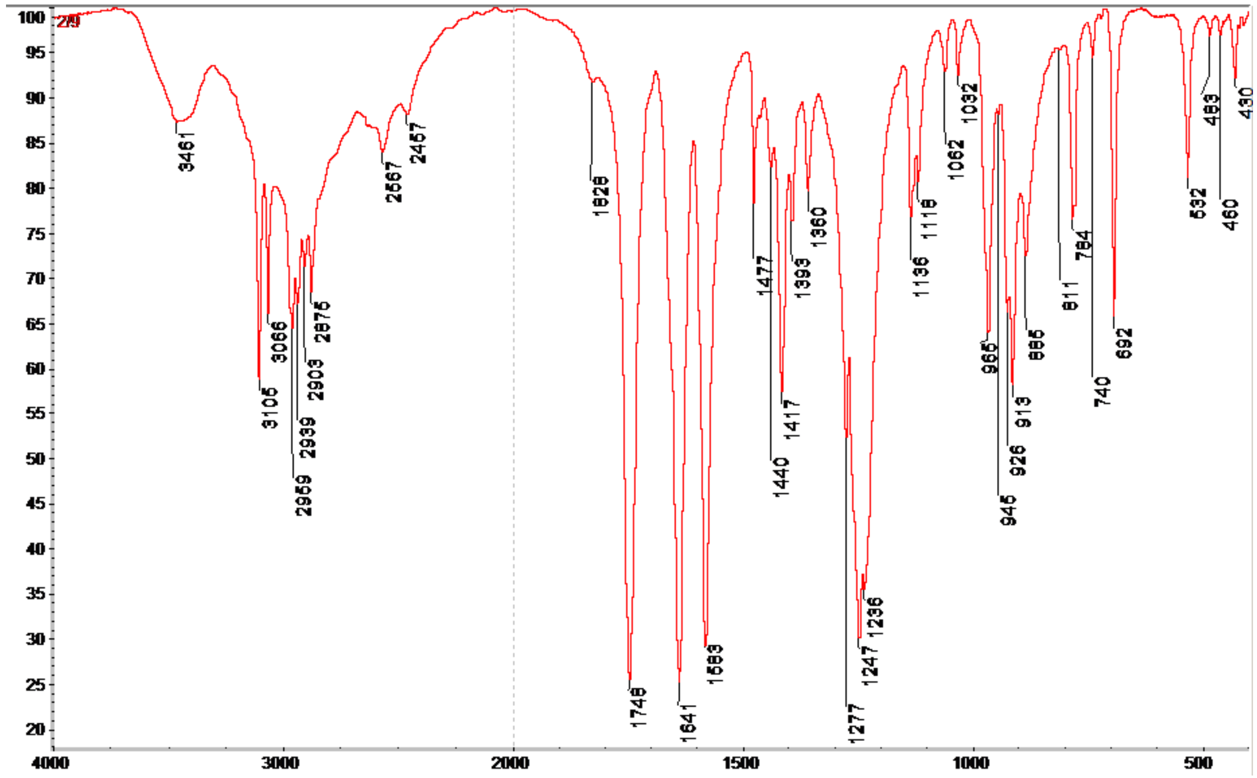

Figure 6S. IR-spectrum (KBr, cm^{-1}) of n-monobutyl ester of chelidonic acid (**3**)

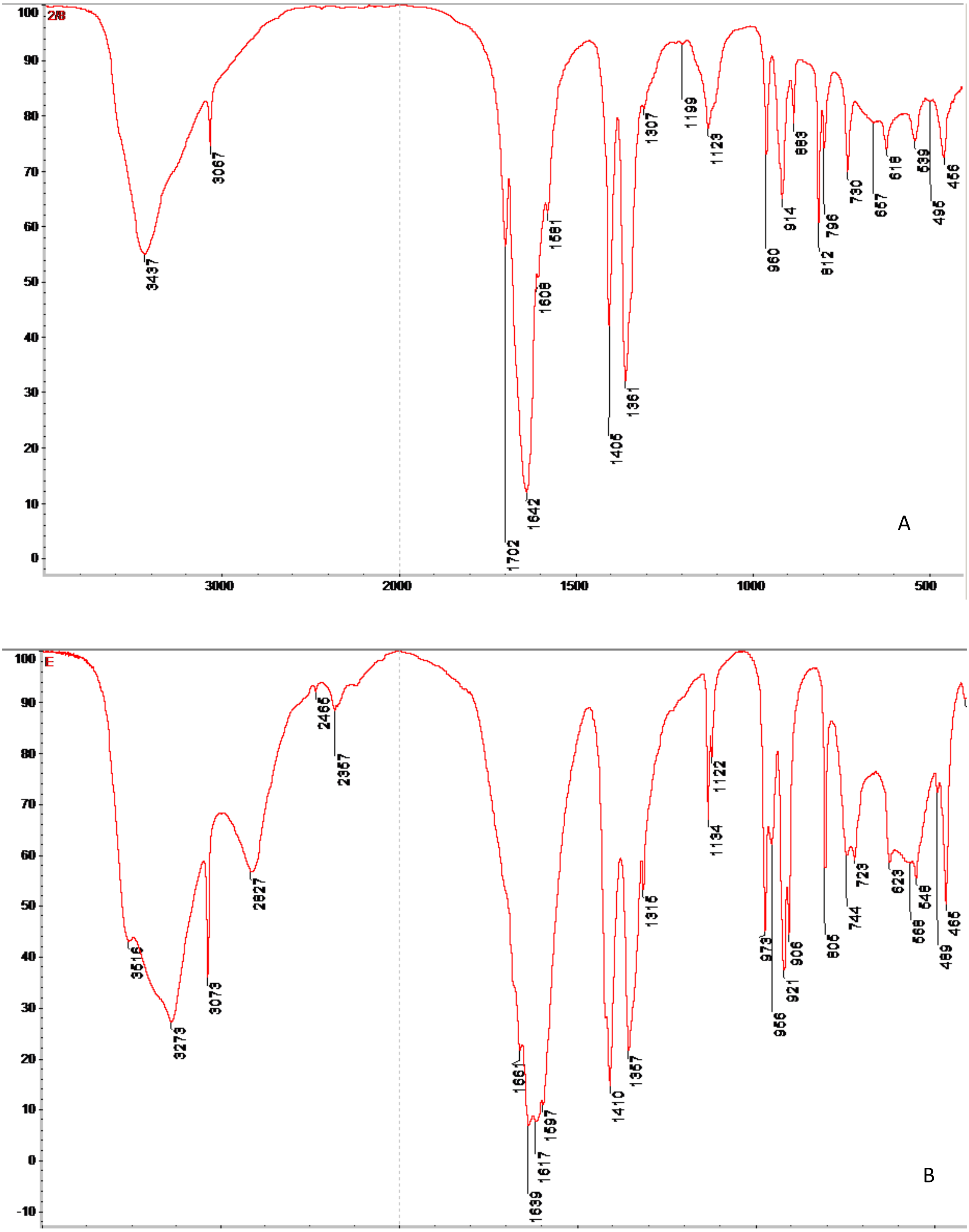

Figure 7S. IR-spectrum (KBr, cm^{-1}) of chelidonic acid **1** (A) and $[Ca(ChA)(H_2O)_3]$ **4** (B).
